# Risks to the delivery of essential nutrition services in Eastern and Southern Africa in the context of the COVID-19 pandemic

**DOI:** 10.11604/pamj.supp.2022.41.2.29081

**Published:** 2022-05-02

**Authors:** Grainne Moloney, Marjorie Volege, Fanuel Odhiambo, Hana Bekele, Mara Nyawo, Kudakwashe Chimanya, Christiane Rudert, Adelheid Onyango

**Affiliations:** 1 UNICEF, East and Southern Africa Regional Office, Emergency Hub for East and Southern Africa, Nairobi, Kenya,; 2 WHO Emergency Hub for East and Southern Africa, Nairobi, Kenya,; 3 WHO Regional Office for Africa, Cité du Djoué, Brazzaville, Republic of Congo

**Keywords:** Nutrition, child nutrition, nutrition, COVID -19

## Abstract

**Introduction:**

without timely action, the global prevalence of child wasting could rise by a shocking 14.3% as a result of disruption of nutrition services by fear, stigma, and various government restrictions to curb COVID-19. Therefore, timely action should be emphasized to ensure continued provision of essential health and nutrition services such as vitamin A supplementation, timely identification and treatment of wasting, provision of micronutrients, and promotion of improved infant and young child feeding (IYCF) in the region.

**Methods:**

this study analyzed the routine nutrition data from HMIS, comparing continuity of essential nutrition services in the region before and during COVID-19. Two online questionnaires were also administered to UNICEF staff in all the 21 ESA countries in May and June 2020.

**Results:**

the Eastern and Southern Africa (ESA) region experienced reduced coverage of vitamin A supplementation among children 6-59 months, while wasting treatment recorded a mixed picture with a 14% overall decline in new admissions, but some countries also reflecting increases. Compared to 2019 there was an increase in the number of mothers and caregivers reached with counselling for improved IYCF. All the countries adopted the revised nutrition programming guidelines in the context of COVID-19.

**Conclusion:**

the impact of COVID-19 to the health and nutrition wellbeing of children and women can't be underestimated. Countries in the region should strive to continue providing essential nutrition services while protecting children and women against the spread of COVID-19. Necessary response measures should be established to build resilience in the health and nutrition sectors to cope with the impact of COVID-19.

## Introduction

By the end of 2019, 690 million people in the world were exposed to severe levels of food insecurity and hunger, which increased by 10 million from 2018, and by nearly 60 million in five years [1]. According to the most recent global estimates of maternal and child malnutrition, 154 million women of reproductive age are underweight, 144 million children under 5 suffer from stunting, and 47 million children under 5 suffer from wasting [2]. The hungry are most numerous in Asia, but in percentage terms, Africa is the hardest hit region and becoming more so, with 19.1 percent of its people undernourished, which includes malnutrition in its multiple forms of stunting, wasting, overweight and micronutrient deficiencies [1]. It is projected that by 2030 Africa will be home to more than half of the world´s chronically hungry. According to the 2020 Joint Malnutrition Estimates, released prior to the pandemic, there were more than 26 million stunted children, and an estimated 10.7 million wasted children including 2.6 million severely wasted children in the 21 countries of Eastern and Southern Africa region (ESA) [2].

The unpredictable evolution of the novel Coronavirus (COVID-19) pandemic poses a significant risk to nutrition outcomes in low- and middle-income countries (LMICs) [3]. Reduced coverage of health and nutrition services associated with the COVID-19 pandemic and a projected two-fold increase in the number of people facing acute food insecurity by the end of 2020 are expected to contribute to increased malnutrition [4]. In the Eastern and Southern Africa Region (ESAR), the pandemic was superimposed on the existing triple threats to food security of drought, floods and desert locusts.

It is estimated that the combination of restrictive measures, disruption of movements, and moderate disruption of food supply chains will result in a 7.9% decrease in gross national income (GNI) per capita will be associated with a projected 14.3% increase in child wasting. This increase, together with the reduction in coverage of essential child health and nutrition services, is projected to result in increased child mortality with an estimated additional 10,000 child deaths per month, and over half of these in sub-Saharan Africa [5]. These projections emphasize the crucial need for actions to ensure continued provision of lifesaving preventive and curative health and nutrition services such as vitamin A supplementation, treatment of wasting, provision of micronutrients, and promotion of improved infant and young child feeding practices especially for the most vulnerable populations in ESA.

### Response measures

To support the continuity of essential nutrition services delivery, several guidance materials were developed at the global and regional levels covering five urgent actions, namely: identification and management of wasting, maternal infant and young child nutrition, micronutrient supplementation, nutrition information management and surveillance, and school nutrition in the context of COVID-19. All countries in the ESA region were supported to adapt the guidance materials based on country-level capacity for program implementation to ensure that: a) essential nutrition services continue to be delivered and used; b) ensure that services adhere to infection prevention and control (IPC) measures to minimize the risk of COVID-19 transmission; c) ensure that no child requiring nutrition services is left behind, especially the most vulnerable.

## Methods

The various methods and sources to assess different aspects of nutrition program adaptations and service coverage are outlined below.

### Adaptation of nutrition programs to COVID-19 context

To understand the extent of COVID-19 related adaptations to nutrition programming at country level, UNICEF ESARO administered two online surveys in May and June 2020 to all 21 countries in the Eastern and Southern Africa subregion. The surveys aimed to: a) assess progress in the adoption of COVID-19 guidance; b) identify key research and learning opportunities; c) understand the response measures put in place at national level to support continuity of nutrition programming; d) the estimated level of disruption (if any) to specific nutrition interventions. The survey questionnaire was completed by UNICEF staff in consultation with Ministry of Health staff.

### New severe acute malnutrition (SAM) treatment admissions

The data on the trend of new admission for severe wasting treatment and vitamin A supplementation coverage were obtained from Health Management Information System (HMIS). The data on new admissions for severe wasting treatment were monthly from January to December for 2017 to 2019 while data for 2020 were up to June 2020. HMIS data on new admission for severe wasting treatment were obtained from only 17 out of 21 ESA countries, therefore four countries whose data were not obtained were excluded in trend analysis.

### Vitamin A supplementation

The HMIS data on vitamin A supplementation were both by semester and monthly from January to December for 2017 to 2019 data while data for 2020 were up to June. VAS trend analysis only included 14 out of 21 ESA countries, therefore 7 countries whose data were not obtained were excluded in the trend analysis. Among the seven countries, three countries do not usually have data available and are not included for any year shown, two countries did not have data available for 2020 and their data has therefore been removed for previous years to allow for comparison, and two countries (Tanzania and Burundi) that usually have campaign data available have not conducted any VAS campaign this year or do not have their data available yet and therefore do not have any data to contribute so far in 2020.

### Infant and young child nutrition

The data on the number of mother or caregivers reached with IYCF counselling or messages were obtained from routine reports from the 21 ESA countries. The data was monthly from January to May 2019 and 2020.

### Nutrition information system

To understand the adoption of nutrition guidance and functionality of nutrition information systems in the context of COVID-19, UNICEF administered two online surveys in May and June 2020 to all the 21 countries in the ESA subregion. The questionnaire was completed by UNICEF staff from the countries in consultation with ministry of health counterparts.

## Results

### Adoption of COVID-19 nutrition guidance

Results from the two UNICEF questionnaires illustrated that all the 21 countries in the Eastern and Southern Africa subregion have adopted the revised programming guidance to varying degrees, with 14 developing country specific guidance.

### Management of wasting (severe acute malnutrition)

**Figure 1:**
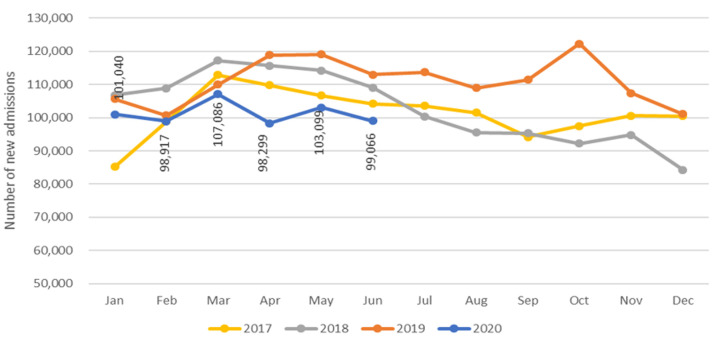
regional trends in admissions for treatment of severe acute malnutrition (preliminary data from country Health Management and Information System, may be subject to revision, data from 17 of 21 countries)

Available data from routine information systems has shown that the subregion has seen an overall decline in the number of new admissions to treatment programs for severe wasting over the first half of 2020 compared to the same time period in 2019. Comparing April, May and June 2020 to the same time in 2019 reveals a 14% reduction in new admissions to treatment of severe wasting. Trends have varied by country, with some countries showing no or limited disruption to services compared to previous years, a decline in service uptake reported from other countries, while a few countries do not have recent data available. Figure 1 presents aggregated data from 17 countries across the ESA region, illustrating that following the initial phase of the pandemic, the number of children treated for severe wasting reduced and continues to remain below the numbers seen during the same period for the past three years (data shown for 17 countries for all years). Despite the overall drop, the level of disruption varied between countries and some countries - particularly those in the Horn of Africa - have seen an increase in admissions, while others have seen a drop that is not, in all cases, attributable only to the effects of COVID-19.

### Vitamin A supplementation

**Figure 2:**
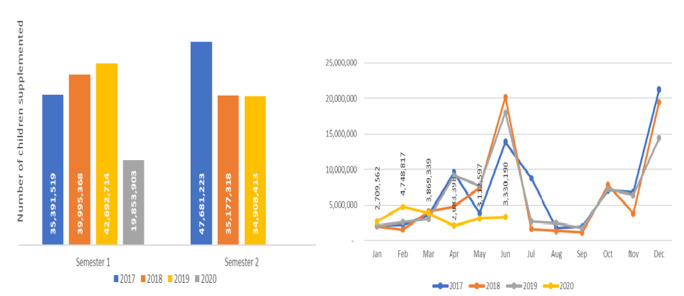
regional trends for Vitamin A supplementation, by semester and by month (preliminary data from country Health Management and Information System, may be subject to revision, data from 14 of 21 countries)

Figure 2 highlights the concerning decline in the number of children receiving VAS in 2020 as compared to the three previous years. In semester 1 (January-June) 2020, under 20 million children aged 6-59 months received their first dose of VAS as compared to over 42 million in semester 1 of 2019. Figure 2 shows data for 2020 for 14 countries (Eritrea, Eswatini, Ethiopia, Kenya, Madagascar, Malawi, Mozambique, Namibia, Rwanda, Somalia, South Sudan, Uganda, Zambia, Zimbabwe). Three countries do not usually have data available and are not included for any year shown, two countries did not have data available for 2020 and their data has therefore been removed for previous years to allow for comparison, and two countries that usually have campaign data available have not conducted any VAS campaign this year or conducted their first campaign in semester 2 (Tanzania and Burundi respectively). It should be noted that some countries that are contributing data from routine supplementation services also usually support VAS through campaigns and therefore their coverage is reduced this year compared to previous years (Kenya, Malawi and Madagascar). Campaigns are re-starting to follow the June 2020 GAVA guidance, and have been conducted in Zambia, Burundi and Namibia in July 2020, therefore their data will contribute to semester 2 coverage.

### Infant and young child nutrition

**Figure 3:**
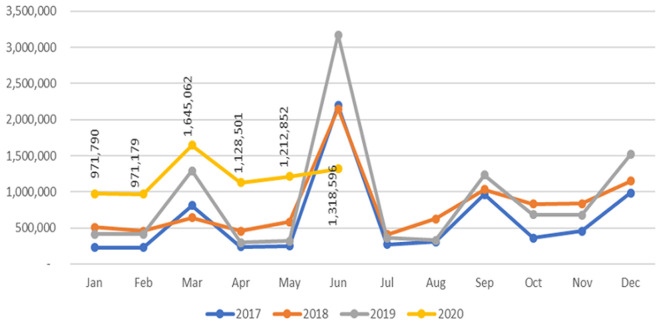
progress on caregivers of children 0-23 months reached with improved infant and young child feeding counselling messages

The routine data showed that while VAS coverage and new admissions for severe wasting treatment declined, the number of caregivers of children aged 0-23 months reached with counselling and messages on improved infant and young child feeding (IYCF) practices increased in the first half of 2020 (January to June) compared to 2019 (January - June) due to scaled-up efforts to protect and promote breastfeeding in the face of the pandemic as shown in Figure 3.

### Nutrition information systems

As reported in the online survey, all countries except Eritrea, reported that routine nutrition information systems were still functioning from the onset of the pandemic. Six countries reported that adaptations have been made to the system, including digital reporting to speed up data availability (Rapid Pro/KoBo Collect), weekly reporting instead of monthly, introduction of additional data elements and indicators, and following up data reporting closely by phone through calls or WhatsApp messaging.

## Discussion

The health sector in the region provides essential services to citizens which include provision of maternal and antenatal care and nutrition services among others. The continued spread of COVID-19 is likely to impact negatively on the provision of these services as focus and efforts are shifted towards controlling its spread. Due to the anticipated disruption of nutrition services, UNICEF and WHO have been supporting countries in Eastern and Southern Africa to adapt their nutrition programs, based on the revised global and regional guidance, to ensure the continuity of preventive and curative essential health and nutrition services. The core nutrition services include management of wasting, maternal infant and young child nutrition counselling, micronutrient supplementation and nutrition information management.

Vitamin A supplementation (VAS) is an essential nutrition intervention, and all children aged 6 to 59 months including those who are suspected or confirmed to have COVID-19 should continue to receive VAS twice a year. Due to the risk of transmission of the COVID-19 virus within communities and between health workers and other community members, modified VAS strategies and administration protocols were adopted to include appropriate infection protection and control (IPC) measures. The initial guidance from the Global Alliance for vitamin A (GAVA) recommended suspension of campaigns that involve mass gatherings, however in June 2020 this was up-dated in line with WHO guidance for immunization campaigns, to recommend that the delivery of VAS through mass campaigns should be considered based on a risk-benefit analysis [6]. Review of the available routine nutrition information from across the region reveals that the pandemic is having an effect on the continuity of essential nutrition services. Vitamin A supplementation (VAS) is the most severely disrupted intervention, and less than half the number of children have received the recommended dose of vitamin A in the first half of 2020 compared to the same time in 2019. Vitamin A supplementation has been severely affected due to the suspension of campaigns and child health days, underscoring the need to embed delivery of VAS into the routine health delivery system to protect it against such shocks in the future.

Management of wasting remains a core programming area for nutrition. Prior to COVID-19, the region was expecting over 2.5 million severely wasted children in 2020 based on the Joint Child Malnutrition Estimates (JME) 2020 [2]. To ensure continuity of lifesaving nutrition services for the early detection and treatment of wasting in anticipation of increase in the prevalence of wasting among children, 9 out of 21 countries in the ESA region have initiated the use of family mid-upper arm circumference (MUAC) while 4 countries have delocalised treatment of uncomplicated wasting to the community level through community health workers. In addition, 6 countries planning to delocalize treatment of wasting. Countries should prioritize these adaptations to ensure critical lifesaving nutrition services reach children in need while minimizing the risk of infection among health care workers and children. The ESA region adopted a no-regrets approach by projecting a 25% pandemic-attributable increase in the regional burden of child wasting in preparedness for the potential impacts of COVID-19.

Suspension of child health days and child health weeks in several countries, unavailability of Personal Protective Equipment (PPE) for routine child health service delivery and low demand for child health services due to the fear of contracting COVID-19 coupled with lockdowns that have resulted in limited mobility of mothers and caregivers may further contribute to decline in new admissions for severe wasting treatment. While the pandemic is causing some level of disruption, it is not the only factor affecting admission trends. Other factors affecting the decline in admissions include supply chain challenges and insecurity, while a change in admission criteria and continued program scale-up and expansion are among the factors affecting the higher admissions in the horn.

Promoting, protecting and supporting breastfeeding and age-appropriate complementary feeding remains a priority in the ESA region [7]. The early issuance of a joint UNICEF, UNHCR, WHO and WFP statement on Infant and Young Child Feeding in the context of the COVID-19 Pandemic in Eastern, Central and Southern Africa in March 2020 [8] was instrumental in ensuring countries had clear guidance on the importance of initiation and continuation of breastfeeding even if the mother was suspected or confirmed with COVID-19. The reduction in June is associated with the suspension of Child Health Day campaigns in many countries as previously described for VAS. Adoption of the use of various digital platforms for capacity building of health workers as well as use of various communication for development approaches and communication channels, has proven effective in reaching mothers with key IYCF messages. The increase in the number of caregivers reached with IYCF counselling and messages was attributed to the increased use of remote communication channels, including digital channels such as short text messaging (SMS). This was deemed essential to counter the increased flow of misinformation around risks of breastfeeding in the context of COVID-19. While the routine data shows an increase in coverage of IYCF counselling and promotion, the effects of COVID on feeding practices and maternal and young child diets is yet unknown, and UNICEF is preparing to undertake analysis and research on this.

Through routine HMIS, ministries of Health and partners have continued to collect routine data on nutrition service delivery and uptake as well as treatment outcomes. Some countries adopted innovative approaches with digital solutions to further strengthen the routine system. Routine information systems have been crucial to enable monitoring of the impact of the pandemic on delivery of essential services.

As the negative socio-economic effects of the pandemic mount and if the region experiences a continued decrease in access to core nutrition services, the risk of increased numbers of undernourished children across the region is real. The reduction in VAS in 2020 is also likely to contribute to an increase in child morbidity and mortality [4,9]. This disruption of essential nutrition services threatens to reverse the gains made in recent years in reducing the burden of malnutrition and child mortality, at a time when most countries in the Eastern and Southern Africa region are far from meeting global World Health Assembly nutrition targets by 2025 [10]. To mitigate such impacts in the health sector and subsequently to protect nutrition outcomes of vulnerable women and children, governments in the region should prioritize the delivery of core preventive and treatment nutrition actions as central to the national COVID-19 response.

## Conclusion

The negative impacts of COVID-19 are of great concern in the region. The potential detrimental effects to the health and nutritional wellbeing of children cannot be underestimated. Countries in the region should continue to implement recommended adaptations to nutrition programming to ensure the continued provision of essential nutrition services, and all stakeholders should combine efforts to implement the five urgent actions identified to protect children´s right to nutrition in the COVID-19 pandemic [11]. To improve vitamin A supplementation (VAS) coverage, countries should explore the possibility of delivering VAS through routine health and nutrition services while adhering to IPC measures. Delivery of VAS through mass campaigns could also be considered based on local factors. ESA countries should begin planning for intense VAS distribution to enable timely mass campaigns and resumption of VAS distribution as soon as deemed possible by ministries of health. Efforts should be made to improve access to treatment of severe wasting by strengthening the capacity of mothers and caregivers to detect or monitor nutrition status of their children using the mid-upper arm circumference (MUAC) tapes. The community health workers (CHWs) should also be trained on the treatment of uncomplicated wasting at the community level. To maintain good access to IYCF counselling and messages, public awareness on protection, promotion and support for appropriate and safe feeding of children should be intensified using context-appropriate channels. Countries in the ESA subregion should strengthen their monitoring and information systems for vitamin A supplementation, child wasting, infant and young child feeding and school feeding using innovative tools such as mobile technologies, thus enabling timely evidence-based decision-making.

### What is known about this topic

- Continuity of essential nutrition services improves maternal and child nutrition outcomes;
- East and Southern Africa region still grapple with the negative effects of malnutrition among children under the age of five and women.

### What this study adds

- COVID-19 disrupted essential nutrition services resulting in decline in vitamin A supplementation and access to treatment of severe wasting;
- The use of innovative communication channels such as SMS and WhatsApp effective to reach mothers or caregivers with key IYCF messages;
- Most countries in the ESA region adopted the revised nutrition programming guidance in the context of COVID-19.
